# Global-local visual processing impacts risk taking behaviors, but only at first

**DOI:** 10.3389/fpsyg.2015.01257

**Published:** 2015-08-31

**Authors:** Stephen Wee Hun Lim, Alexander Y. L. Yuen, Eddie M. W. Tong

**Affiliations:** Department of Psychology, Faculty of Arts and Social Sciences, National University of Singapore, Singapore

**Keywords:** global-local visual processing, Balloon Analogue Risk Task, decision making, risk taking behavior, theory of predictive and reactive control systems

## Abstract

We investigated the impact of early visual processing on decision-making during unpredictable, risky situations. Participants undertook Navon’s (1977) task and attended to either global letters or local letters only, following which they completed the Balloon Analogue Risk Task (BART). It was observed that global-focused individuals made more balloon pumps during the BART (i.e., took more risk), whereas local-focused individuals took less risk, albeit only initially. The theory of predictive and reactive control systems (PARCS) provides an excellent account of the data. Implications and future directions are discussed.

## Introduction

We make decisions all the time in response to our ever-changing environment. We usually experience the physical world by first acquiring basic perceptual information about it through various sensory channels (e.g., our visual system). This information is often filtered and subsequently routed to be processed at the higher visual areas, and in turn feeds into and shapes complex behavioral responses, such as whether or not to confront or flee from a source of danger (e.g., a snake), involving estimating probabilities of attaining desired outcomes in an unpredictable situation. Risk taking continues to play a critical role in such modern day events as driving, gambling, and engaging in (or deterring) criminal activities. Researchers have studied the role of executive functions (Romer et al., 2009; Kóbor et al., 2015), personality and individual differences (e.g., Lauriola et al., 2005; Bornovalova et al., 2009), and neural activity (Brand et al., 2006; Fecteau et al., 2007; Helfinstein et al., 2014) in risk-taking behaviors, although less attention has been devoted to understanding the impact of rudimentary perceptual processes on decision-making in ambiguous and risky scenarios. Here, we adopted the theory of predictive and reactive control systems (PARCS; Tops et al., 2010, 2014) that provides a primary source of inspiration for our work, which explored whether, and in what way, early global-local visual processing would influence higher-order risk taking behaviors during unpredictable situations.

### Global-local Visual Processing

Fundamental to Gestalt psychology is the view that a whole is qualitatively different than the resultant percept that one might expect by processing only its parts. Under this view, wholes are formed prior to the perceptual analysis of their properties and components in perceptual organization. Navon (1977) proposed that perceptual processing begins with global structuring, and later shifts toward finer analyses. This proposal was termed as the global precedence hypothesis, and has been tested by studying the perception of hierarchical patterns in which larger figures are constructed by suitable configurations of smaller figures. An example is a set of large letters constructed from the same set of smaller letters having either the same identity as the larger letter or a different identity (see Förster and Higgins, 2005; Lim and Goh, 2013). The larger letter is considered a higher-level unit, whereas the smaller letters are considered lower level units. Specifically, properties of the higher level unit are considered more global than properties of the lower level units by virtue of their positioning in the hierarchical structure. In a typical experiment, individuals are presented with such stimuli and instructed to identify the larger (i.e., global) or the smaller (i.e., local) letter. Broader perceptual scope is indicated by relatively faster responses to letters presented as global targets, whereas narrower perceptual scope is reflected in relatively faster responses to the same letters presented as local targets. In this context, local-focused individuals are said, colloquially speaking, to have “missed the forest for the trees.”

### The Theory of Predictive and Reactive Control Systems

Tucker and his colleagues (Tucker and Williamson, 1984; Derryberry and Tucker, 1994) proposed a useful evolutionary framework for understanding global-local processing in a variety of contexts (see Friedman and Förster, 2010, for a comprehensive review). The broad view is that under menacing circumstances, constricted attention is useful because it enables the individual to focus on the immediate problem and locating potential solutions. By the same token, when the individual moves away from the dangerous situation, broadened (rather than constricted) attention is useful because it enables the individual to form new mental representations of his or her surroundings and acquire novel resources. This view has been supported by studies which revealed that individuals are less accurate and/or take longer to detect peripheral visual targets during anxiety-inducing situations (e.g., Callaway and Dembo, 1958; Weltman et al., 1971; Reeves and Bergum, 1972; Burke et al., 1992).

According to the model, there exist two types of brain systems that have developed over evolutionary time. One type is guided by context models that are built in long-term memory based on the predictability of the environment. This type of brain systems controls cognition and behaviors in highly predictable environments. The other type of brain systems, in contrast, controls cognition and behaviors in unpredictable environments, in which context models cannot effectively develop nor function. Tops et al. (2010) expanded this original model, adding the idea of a narrow focus in space and time on not just avoiding punishment but also attaining reward within the reactive system. In that regard, temporary feedback from environmental stimuli guides behavior reactively in such environments, in which stimuli—not just negative (e.g., dangerous) ones but, according to the revised model, positive (e.g., rewarding) ones also—are close in psychological space and time and require urgent attention. Contrastingly, there is less urgency and a more global focus in space and time when behavior is guided proactively in a feed-forward fashion by internal models in high-predictable (as opposed to low-predictable) environments.

Together, these two types of systems undergird the theory of PARCS (see, also, Tops and Boksem, 2011; Tops et al., 2013, 2014). The view is that reactive control systems applying feedback-guided control possibly encourage the individual to explore new surroundings, and gather information that supports the development of new internal models (and the updating of existing ones). For instance, learning a novel but predictable task first involves the reactive control system to deal with novelty, but once internal working models are developed, the predictive control system takes over, where control is now more habitual. Of particular relevance for the present study is the tenet that predictive control is associated with a global attentional focus in psychological space and time and a behavioral focus on obtaining gains (i.e., “promotion focus”), whereas reactive control is associated with a local (narrower) attentional focus in space and time and a behavioral focus on preventing loss (i.e., “prevention focus”). Specifically, inducing a global focus involves processing in the predictive control system which triggers a bias toward a promotion focus, whereas inducing a local focus initiates processing in the reactive control system which triggers a bias toward a prevention focus (see, also, Förster and Higgins, 2005, for a discussion on an analogous reciprocal association between global/local processing and promotion/prevention focus). Accordingly, the PARCS theory predicts that during ambiguous situations in which rewards are not guaranteed, global-focused subjects would take more risk to pursue new gains, as opposed to local-focused individuals who would take less risk to avoid losses.

### The Present Study

We manipulated global-local visual processing by applying Navon’s (1977) task, and recorded the extent to which it impacted participants’ tendencies in taking risks. We hypothesized that participants who attended to global letters would be more inclined to take risks than would participants who attended to local letters. In particular, we examined the time course of this proposed effect. We wish to emphasize the importance of considering the activation trajectory of global-local processing. Rudimentary sensory/perceptual information is short-lived and, on its own (without deeper processing), relatively less likely to be retained in long-term memory (Craik and Lockhart, 1972). To this end, any effects arising from the present manipulation can dissipate over time. To explore this possibility, we segmented the experimental trials of the present Balloon Analogous Risk Task (BART; see Materials and Methods for details) into blocks, and hypothesized that risk taking behaviors (BART responses) arising from the global visual processing condition would differ from responses in the local visual processing condition but only in the earlier, rather than the later, blocks.

## Materials and Methods

### Participants

Forty-eight undergraduates (18 were male; 30 were female) from the National University of Singapore participated to fulfill course credit requirements, although two female participants did not complete the study and their data were subsequently excluded from the analyses. Participants were randomly assigned to the global or local visual processing condition.

### Procedures

The experiment was conducted with no more than 10 participants within a single session. Each session took about 25 min. Upon arrival, each participant was randomly assigned to a personal computer with a regular response time box attached comprising of six equally-spaced buttons through which responses were made. The extreme right button (red in color) and extreme left button (blue in color) were designated for responses, respectively. Participants were separated by partitions throughout the whole session, so that they could not communicate with, nor view the computer screens and activities of, other participants. This way, any competition between participants was prevented.

**FIGURE 1:**
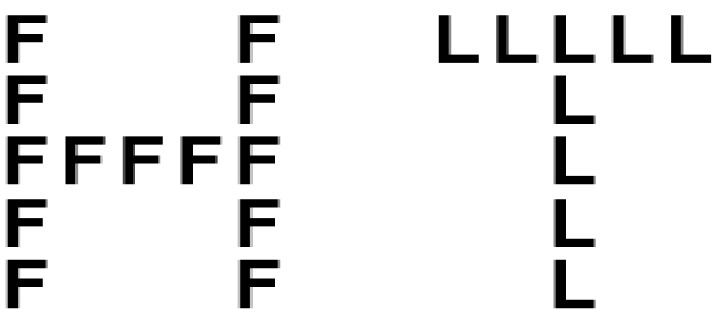
**Examples of composite visual letters used in the present experiment**.

Participants indicated their consent to participate in the experiment before embarking on it, which consisted of two tasks: Navon’s task and BART. The Navon’s task was administered via *DirectRT* (Jarvis, 2004). Participants were shown a series of global letters that were composed of local letters. Following Förster and Higgins (2005), each global letter was approximately 2.1 cm × 2.1 cm, while each local letter was approximately 0.4 cm × 0.4 cm. Local letters were arranged on an imaginary 5-letter × 5-letter grid for presentation. The letters *H* and *L* were designated as targets. The targets were presented either as global letters (an *H* made of *F*s, an *H* made of *T*s, an *L* made of *F*s, and an *L* made of *T*s) or local letters (an *F* made of *H*s, an *F* made of *L*s, a *T* made of *H*s, and a *T* made of *L*s). Samples appear in Figure 1.

At the start of each trial, participants were presented with a fixation cross at the centre of the screen for 500 ms. Following which, one of the eight composite letters appeared, substituting the fixation; each letter had an equal chance of being presented. Participants were told to press the blue button if the composite stimulus contained the letter *L*, and the red button if it contained the letter *H*, as quickly as possible but not compromising accuracy. Participants in the global condition saw composite stimuli in which the target letters were always global letters, whereas those in the local condition saw stimuli in which the target letters were always local letters. Each participant completed 12 practice trials, followed by 18 test trials.

Immediately after completing Navon’s task, participants underwent the BART which, among tasks investigating the underlying factors of risky decision making and real-world risk taking, is one of the most widely used (Lejuez et al., 2002). Participants pumped a balloon, where each pump is associated with either a reward gain or an unknown probability that the balloon may pop. Following each successful pump, participants could either stop and collect the reward accumulated thus far, or continue pumping. If the balloon pops, all of the accumulated reward is lost. The probability of a balloon pop increases with each successive pump, but the probability structure that governs the balloon pops is not divulged to participants. Thirty BART trials were administered in succession without breaks in between. Participants were debriefed after they completed their sessions.

## Results

We employed Hierarchical Linear Modeling (HLM; Raudenbush and Bryk, 2002) to examine the effects of global-local visual processing on risk taking. HLM is suitable for our purpose because it allows for estimation of within-participants effects (i.e., the temporal trajectories of risk taking) nested within between-participants effects (i.e., visual processing). Following Lejuez et al.’s (2002) recommendations, we computed the mean adjusted number of pumps (MPA) which refers to the average number of pumps across all the trials excluding those in which balloons had popped. Specifically, to test the temporal trajectory of visual processing, the BART trials were parsed into three blocks of 10 trials each. Three sets of MPA scores were computed by averaging the scores within the first, second, and final 10 trials. At Level 1 of the HLM analysis, we entered *MPA* as the dependent variable and *block* as the predictor. *Block* is a time-based variable in which the first, second, and final 10 trials were coded as 1, 2, and 3, respectively. Hence, the relationship between *MPA* and *block* indicates the linear rate of change of risk taking across the three blocks. At Level 2, we entered *processing* (global was coded as 1; local was coded as 0) to predict both the overall average of *MPA* across all 30 trials and the relationship between *MPA* and *block*. All Level 2 random effect terms were specified in the present model.

A significant effect of visual processing on the average level of MPA across all 30 trials emerged, *B* = 12.16, SE = 5.55, *p* = .034. Across all 30 trials, participants in the global condition produced, on average, more pumps than did those in the local condition (see Table 1). There was also a linear effect of block, *B* = 2.46, SE = 0.93, *p* = .011, indicating that participants across both conditions produced more pumps from the first 10 trials to the final 10 trials. Paired-sample *t*-tests revealed that the number of pumps in the 1st block did not differ from that in the 2nd block, *t*(47) = 1.28, *p* = .21, while significantly more pumps were made from the 2nd block to the 3rd block, *t*(46) = 3.55 *p* = .001.

**TABLE 1:** **Mean adjusted number of pumps (MAP; standard deviations in parentheses) as a function of global-local visual processing and BART blocks**.

	**Block 1**	**Block 2**	**Block 3**	**Overall**
Global	36.25 (14.98)	34.88 (13.67)	35.78 (12.84)	35.86 (13.42)
Local	28.09 (16.88)	32.97 (15.43)	37.76 (17.92)	32.43 (15.26)
Overall	32.35 (16.27)	33.97 (14.40)	36.75 (15.39)	34.22 (14.27)

More important, *process* significantly moderated the relationship between *MPA* and *block*, *B* = –4.60, SE = 1.88, *p* = .019. For participants in the global condition, there was no change in the number of pumps from the 1st to the 2nd block, *t*(23) = 0.72, *p* = .48. At first blush, a change in the number of pumps from the 2nd to the 3rd block emerged, but the difference was only marginally significant, *t*(22) = 1.95, *p* = .06. In contrast, in the local condition, the number of pumps administered increased significantly from the 1st to the 2nd block, *t*(21) = 2.63, *p* = .016, and from the 2nd to the 3rd block, *t*(21) = 2.75, *p* = .012. Comparing across conditions within each block, the global condition was found to produce more pumps than did the local condition in the 1st block, *t*(44) = 1.74, *p* = .04, as directionally predicted. There was no difference between the processing conditions in the 2nd block, *t*(44) = 45, *p* = .66, nor in the 3rd block, *t*(43) = 41, *p* = .68.

## Discussion

The present study investigated the impact of rudimentary global-local visual processing on decision-making in an unpredictable, risky situation. We specifically acknowledged the possibility that sensory/perceptual-level effects are relatively less durable, which may weaken with time. Thus, the hypothesis was that BART responses from individuals who earlier processed global letter information would differ from those who did local letter information, but this difference would obtain only during the earlier BART trials. The present data provided support for this hypothesis. Participants who attended to global letters consistently made around 35 pumps throughout the 30 BART trials. In contrast, participants who attended to local letters appeared, on average, relatively more circumspect in making pumps initially (around 28 pumps in the 1st block), but their pump count increased with blocks and finally became on par with that of the global-processing group by the 3rd block. An insight arising from the data is that the effects appear to be driven primarily by local-focused participants, suggesting that a global focus may, otherwise, be more or less the default mode of focus.

The theory of PARCS (Tops et al., 2010, 2014; see Introduction) serves as an excellent anchor for understanding our findings. Recapitulating, the brain comprises of two systems that are associated with predictive and reactive control, respectively. The predictive system handles high-predictable situations in which behavior is guided in a feedforward fashion, whereas the reactive system deals with low-predictable situations in which temporary feedback from environmental stimuli provide feedback to guide behavior (see, also, Kóbor et al., 2015). The crucial idea is that predictive control relates to a global attentional focus in psychological space and time, and is promotion-focused, whereas reactive control relates to a local (narrower) attentional focus in that space and time, and is prevention-focused. Based on this theory, global-focused subjects take more risk to pursue new gains, whereas local-focused individuals take less risk to avoid losses.

In our study, global-focused individuals were indeed more liberal in making balloon pumps as compared to local-focused individuals who were significantly more conservative in making pumps at the beginning. Taking into account that a global (rather than a local) focus appears to be the default mode of focus (as discussed two paragraphs earlier), the interpretation is that, for as long as the transient effects of global-local visual processing persisted, local-focused individuals are better able to exert inhibitory control which involves overriding one’s predominant response tendencies—in this case, their global focus (see Schmeichel et al., 2011, for a discussion on inhibitory self-control). When the visual processing effects dissipated, individuals are likely to return to their global focus, as evidenced by local focused participants’ liberal behaviors in making balloon pumps in the later trials. Future research should test this interpretation directly.

Finally, it is noteworthy that positive emotional states and implicit affective cues lead to a global focus, whereas negative emotional states and implicit affective cues lead to a local focus, at both the perceptual and conceptual levels (see Friedman and Förster, 2010, for a comprehensive review). Future studies can investigate whether, and to what extent, affect mediates the relationship between early visual processing and risk taking behaviors. This will, in turn, enable a fuller understanding of the way in which early perceptual processing impacts higher-order cognition and risk-taking decisions over psychological time.

### Conflict of Interest Statement

The authors declare that the research was conducted in the absence of any commercial or financial relationships that could be construed as a potential conflict of interest.

## References

[B1] Bornovalova M. A. Cashman-Rolls A. O’Donnell J. M. Ettinger K. Richards J. B. deWit H. (2009). Risk taking differences on a behavioral task as a function of potential reward/loss magnitude and individual differences in impulsivity and sensation seeking. Pharmacol. Biochem. Behav. 93, 258–262. 10.1016/j.pbb.2008.10.023 19041886

[B2] Brand M. Labudda K. Markowitsch H. J. (2006). Neuropsychological correlates of decision-making in ambiguous and risky situations. Neural Netw. 19, 1266–1276. 10.1016/j.neunet.2006.03.001 16942857

[B3] Burke A. Heuer F. Reisberg D. (1992). Remembering emotional events. Mem. Cogn. 20, 277–290. 10.3758/BF03199665 1508053

[B4] Callaway E. Dembo D. (1958). Narrowed attention: a psychological phenomenon that accompanies a certain physiological change. Arch. Neurol. Psychiatry 79, 74–90. 10.1001/archneurpsyc.1958.02340010092008 13486983

[B5] Craik F. I. M. Lockhart R. S. (1972). Levels of processing: a framework for memory research. J. Verbal Learn. Verbal Behav. 11, 671–684. 10.1016/S0022-5371(72)80001-X

[B6] Derryberry D. Tucker D. M. (1994). “Motivating the focus of attention,” in Heart’s eye: Emotional Influences in Perception and Attention, eds Niedenthal P. M. Kitayama S. (New York, NY: Academic Press), 167–196. 10.1016/B978-0-12-410560-7.50014-4

[B7] Fecteau S. Pascual-Leone A. Zald D. H. Liguori P. Théoret H. Boggio P. S. (2007). Activation of prefrontal cortex by transcranial direct current stimulation reduces appetite for risk during ambiguous decision making. J. Neurosci. 27, 6212–6218. 10.1523/JNEUROSCI.0314-07.2007 17553993 PMC6672163

[B8] Förster J. Higgins E. T. (2005). How global versus local perception fits regulatory focus. Psychol. Sci. 16, 631–636. 10.1111/j.1467-9280.2005.01586.x 16102066

[B9] Friedman R. S. Förster J. (2010). Implicit affective cues and attentional tuning: an integrative review. Psychol. Bull. 136, 875–893. 10.1037/a0020495 20804240 PMC2933078

[B10] Helfinstein S. M. Schonberg T. Congdon E. Karlsgodt K. H. Mumford J. A. Sabb F. W. (2014). Predicting risky choices from brain activity patterns. Proc. Natl. Acad. Sci. U.S.A. 111, 2470–2475. 10.1073/pnas.1321728111 24550270 PMC3932884

[B11] Jarvis B. G. (2004). DirectRT Research Software (Version 2004) [Computer software]. New York, NY: Empirisoft.

[B12] Kóbor A. Takács Á. Janacsek K. Németh D. Honbolygó F. Csépe V. (2015). Different strategies underlying uncertain decision making: higher executive performance is associated with enhanced feedback-related negativity. Psychophysiology 52, 367–377. 10.1111/psyp.12331 25224177

[B13] Lauriola M. Russo P. M. Lucidi F. Violani C. Levin I. P. (2005). The role of personality in positively and negatively framed risky health decisions. Pers. Individ. Dif. 38, 45–59.

[B14] Lejuez C. W. Read J. P. Kahler C. W. Richards J. B. Ramsey S. E. Stuart G. L. (2002). Evaluation of a behavioural measure of risk taking: the Balloon Analogue Risk Task (BART). J. Exp. Psychol. Appl. 8, 75–84. 10.1037/1076-898X.8.2.75 12075692

[B15] Lim S. W. H. Goh W. D. (2013). Articulation effects in melody recognition memory. Q. J. Exp. Psychol. 66, 1774–1792. 10.1080/17470218.2013.766758 23410265

[B16] Navon D. (1977). Forest before trees: the precedence of global features in visual perception. Cogn. Psychol. 9, 353–383. 10.1016/0010-0285(77)90012-3

[B17] Raudenbush S. W. Bryk A. S. (2002). Hierarchical Linear Models, 2nd Edn. Thousand Oaks, CA: Sage.

[B18] Reeves F. B. Bergum B. O. (1972). Perceptual narrowing as a function of peripheral cue relevance. Percept. Mot. Skills 35, 719–724. 10.2466/pms.1972.35.3.719 4643960

[B19] Romer D. Betancourt L. Giannetta J. M. Brodsky N. L. Farah M. Hurt H. (2009). Executive cognitive functions and impulsivity as correlates of risk taking and problem behavior in preadolescents. Neuropsychologia 47, 2916–2926. 10.1016/j.neuropsychologia.2009.06.019 19560477 PMC2780004

[B20] Schmeichel B. J. Vohs K. D. Duke S. C. (2011). Self-control at high and low levels of mental construal. Soc. Psychol. Personal. Sci. 2, 182–189. 10.1177/1948550610385955

[B21] Tops M. Boksem M. A. S. (2011). A potential role of the inferior frontal gyrus and anterior insula in cognitive control, brain rhythms and event-related potentials. Front. Psychol. 2:33. 10.3389/fpsyg.2011.00330 22084637 PMC3212750

[B22] Tops M. Boksem M. A. S. Luu P. Tucker D. M. (2010). Brain substrates of behavioral programs associated with self- regulation. Front. Psychol. 1:152. 10.3389/fpsyg.2010.00152 21887146 PMC3157933

[B23] Tops M. Boksem M. A. S. Quirin M. IJzerman H. Koole S. L. (2014). Internally-directed cognition and mindfulness: an integrative perspective derived from predictive and reactive control systems theory. Front. Psychol. 5:429. 10.3389/fpsyg.2014.00429 24904455 PMC4033157

[B24] Tops M. Luu P. Boksem M. A. S. Tucker D. M. (2013). “The roles of reactive and predictive behavioral/physiological programs in resilience,” in The Resilience Handbook: Approaches to Stress and Trauma, eds Kent M. Davis M. C. Reich J. W. (New York: Routledge Publishers), 15–32.

[B25] Tucker D. M. Williamson P. A. (1984). Asymmetric neural control systems in human self-regulation. Psychol. Rev. 91, 185–215. 10.1037/0033-295X.91.2.185 6152836

[B26] Weltman G. Smith J. E. Edstrom G. H. (1971). Perceptual narrowing during pressure-chamber exposure. Hum. Factors 13, 99–107. 5550590 10.1177/001872087101300202

